# Predictors of psychotic experiences among adolescents with obsessive–compulsive symptoms: A data‐driven machine learning approach

**DOI:** 10.1002/pcn5.70103

**Published:** 2025-05-01

**Authors:** Yutaka Sawai, Riki Tanaka, Rin Minami, Daiki Nagaoka, Akito Uno, Ayako Okuma, Syudo Yamasaki, Mitsuhiro Miyashita, Atsushi Nishida, Kiyoto Kasai, Shuntaro Ando

**Affiliations:** ^1^ Department of Neuropsychiatry Graduate School of Medicine, The University of Tokyo Tokyo Japan; ^2^ Research Center for Social Science & Medicine Tokyo Metropolitan Institute of Medical Science Tokyo Japan; ^3^ The International Research Center for Neurointelligence (WPI‐IRCN) at the University of Tokyo Institutes for Advanced Study (UTIAS) Tokyo Japan

**Keywords:** data‐driven approach, general population, machine learning, obsessive–compulsive symptoms, psychotic experiences

## Abstract

**Aim:**

Prediction of future psychosis in individuals with obsessive and compulsive (OC) symptoms is crucial for treatment choice, but only a few predictors have been revealed. Although OC symptoms and psychotic experiences (PEs) are common in adolescence, no studies have revealed the predictors of subsequent PEs in adolescents with OC symptoms. We aimed to explore the predictors for subsequent PEs among adolescents with OC symptoms, using a data‐driven machine‐learning approach on an adolescent cohort.

**Methods:**

We used data from a cohort study on the general population of adolescents in Tokyo (*n* = 3171 at age 10). Data were collected at age 10, 12, 14, and 16. We focused on a subgroup of participants who had OC symptoms at age 12. Participants who had PEs at age 10 were excluded. A machine learning method was utilized to explore over 600 potential predictors at baseline, distinguishing between those who had an onset of PEs after age 14 (*n* = 45) and those who never had PEs (*n* = 99).

**Results:**

The predicting model demonstrated a good performance (test area under the curve = 0.80 ± 0.05). Other than known risk factors for PEs, novel predictors of subsequent PEs among adolescents with OC symptoms included: lack of interaction with people of different ages, desire to be like their father in the future, and nonworking of primary caregiver when they were 5 years old. Not sharing their belongings readily with other children was a strong predictor of having no PEs.

**Conclusion:**

Close‐knit family bonds and limited social connections outside the family predict the later PEs among adolescents with OC symptoms.

## INTRODUCTION

Clinical diagnosis of obsessive–compulsive disorder (OCD) is associated with an increased risk of subsequent diagnosis of psychosis.^1^, ^2^ Individuals who were first diagnosed as having OCD were three times more likely to develop psychosis, compared to those without OCD.^1^ It is also known that obsessive and compulsive (OC) symptoms can be found in the high‐risk, prodromal, and premedication stages of schizophrenia.^2^ A meta‐analysis indicated that 30% of patients with schizophrenia had subclinical OC symptoms.^3^

When treating patients with OCD, it is important to detect the underlying psychosis. Specifically, selective serotonin reuptake inhibitors (SSRIs), often the first‐line treatment option for OCD, may exacerbate psychotic symptoms in the prodromal phase of schizophrenia.^4^ Comorbid schizotypal personality is indicative of unfavorable response to standard pharmacological and behavioral treatment for OCD.^5^ “Atypical” OCD, which presents with delusional states, is associated with greater social maladjustment and poor response to treatment.^6^ It is therefore crucial to predict whether people with OCD will develop psychosis because it greatly impacts the choice of treatment.^7^, ^8^ However, there is a lack of understanding regarding the predictors of psychotic symptoms in individuals with OCD because of an overlap between OCD and psychosis in terms of the course of the disorder and the limited number of cases.^9^ A cross‐sectional study revealed that childhood trauma in people with OCD may be related to psychosis,^10^ but other sociocultural predictors are unknown.

Subclinical OC symptoms are prevalent in the general population.^11^ More than one‐quarter of the general population experiences OC symptoms during their lifetime.^11^ Although OCD is diagnosed in just 0.5%–4% of the adolescent general population,^12^, ^13^, ^14^ 8% of the adolescent general population reported subclinical OC symptoms.^15^ A previous study revealed that individuals with subclinical OC symptoms share common neurobiological substrates with clinical OCD.^16^

Psychotic experiences (PEs) are also subclinical symptoms, including hallucinations and delusions.^17^ In the general population of adolescents, the prevalence of PEs was reported as 10%.^18^ PEs during childhood and adolescence were associated with an elevated risk of adult psychotic disorders.^19^ PEs and psychosis have several common risk factors,^20^ suggesting that research on PEs in the general population would be helpful for deeper understanding of psychosis.^21^

Taken together, investigating predictors for PEs from subclinical OC symptoms could potentially contribute to understanding predictive factors for the development of psychosis from OCD. Nevertheless, no studies have revealed the predictors of subsequent PEs among individuals with OC symptoms. Our objective is to explore the predictors for subsequent PEs in adolescents with OC symptoms in a longitudinal population‐based cohort study. It has been challenging to predict the subsequent PEs based only on the contents of OC symptoms.^22^, ^23^ Hence, we looked beyond pathophysiology and focused on psycho‐eco‐social factors during participants' childhoods. We attempted a data‐driven approach to explore predictors from a diverse range of variables, including demographic characteristics, behavioral and psychometric measures, and personality profiles. We used gradient boosted classification modeling and a machine learning algorithm with good interpretability and good performance for large datasets.^24^

## METHODS

### Study design and participants

This study used data from the Tokyo Teen Cohort (TTC) study.^25^ The TTC study is a population‐based longitudinal cohort study to investigate adolescent health and development (*n* = 3171). Adolescents (born between September 1, 2002 and August 31, 2004) and their primary caregivers were randomly recruited from three municipalities (Setagaya‐ku, Mitaka‐shi, and Chofu‐shi) in the metropolitan area of Tokyo when the children were 10 years old. Data were collected at four time points, when the children were at age 10 (T1), 12 (T2), 14 (T3), and 16 (T4) years. The follow‐up rates were 94.8% at T2 (*n* = 3007), 84.1% at T3 (*n* = 2667), and 82.5% at T4 (*n* = 2616).

We focused on a subgroup of participants who had OC symptoms at T2. For the current analysis, participants who had PEs at T1 were excluded, in order to compare those who had new PEs after T3 with those who had never had PEs.

### Measures

#### OC symptoms

OC symptoms were assessed by items derived from the Child Behavior Checklist (CBCL),^26^ which is a caregiver‐answered questionnaire. The answers for each question were chosen from *Not true* (0 points), *Somewhat or sometimes true* (1 point), and *Very true or often true* (2 points). Obsessions were assessed by one question asking caregivers whether their children could not get their mind off certain thoughts. Compulsions were measured by another question asking caregivers whether their child repeated certain acts over and over. The score for OC symptoms was summed up to a total score, ranging from 0 to 4. According to a previous study,^27^ children with one or more points out of four in the total score were defined as having OC symptoms at T2 (*n* = 302).

#### Psychotic experiences

Current PEs were assessed by a self‐report questionnaire. We assessed the five types of PEs (auditory hallucinations, persecutory thoughts, visual hallucinations, thought broadcasting, and special messages through the TV or radio) during the last 2 weeks. We derived five items for each type of PE from Psychosis Screening Questionnaire 9 (PSQ9).^28^ Children answered the questions on a four‐point scale, *No, never*, *Maybe*, *Only once*, and *Twice or more*.

For subsequent analyses, we only included data from children who definitely had PEs (*Only once*/*Twice or more* at least one item at T3 or T4) or who definitely lacked PEs (*No, never* for all items at T3 or T4). This excluded 158 participants with intermediate answers (i.e., those who answered only *Maybe* or both *No, never* and *Maybe*). This resulted in reducing the size of the analyzed population to 144 participants.

#### Candidates of predictors

We acquired all quantitative data at T1 and the Autism‐Spectrum Quotient‐10 (AQ‐10)^29^ adolescent questionnaire from T2. The dataset consisted of 1288 measured and engineered variables, comprehensively including psycho‐eco‐social factors.^25^ To focus on children's mental health and behavior problems, we gathered measurements such as the Strengths and Difficulties Questionnaire (SDQ)^30^, ^31^ and the CBCL.^26^ The SDQ is commonly used for screening emotional and behavioral difficulties in children and young people. The CBCL is a widely used form that allows parents to evaluate and describe their children's emotional and behavioral challenges. Both the SDQ and CBCL are caregiver‐rated scales. Other variables included children's psychological development/health, children's cognitive development, children's physical development/health, perinatal environment, and household environment. Then variables with missing values of <5% were used for machine learning modeling (630 variables). We performed imputation of missing data in predictors using random forest via “missForest.”^32^ The number of trees in the random forest is set to 100.

### Data analysis

In order to explore relevant variables, we used Extreme Gradient Boosting (XGBoost),^24^ a decision‐tree‐based machine learning algorithm, to train the classification models. XGBoost easily suffers from overfitting, owing to the relatively small sample size. Repeated nested cross‐validation was thus utilized to achieve better generalization to independent data and unbiased results.^33^, ^34^

In this study, data were divided into four similarly sized subsamples. A four‐fold stratified cross‐validation (CV) was used, where three folds acted as a training set, and one as a hold‐out test set (the outer loop). The training set was then split into four stratified folds with three training folds, and one hold‐out fold (the inner loop). In the inner loop, CV was performed to find the best hyperparameters. To balance sensitivity (true positive rate) and specificity (true negative rate), Youden's index^35^ was used to optimize the parameters. Youden's index remedied the bias due to imbalance in classes in favor of those who had no PEs at T3 and T4. Tuned parameters included the number of estimators (1, 5, 10, 20, 30, 40, 50, 60, 70, 80, 90, or 100), learning rate (0.1, 0.2, or 0.3), and tree depth (1 or 2). These ranges were set to reduce the risk of overfitting and to make the model easier for interpretation. Training was repeated 100 times in each outer loop, resulting in 100 × 4 unique models. The final classifier was trained on the full training set using the best parameters. Then, the model performance was validated on the hold‐out test set. In each 400 model, the Boruta random forest algorithm^36^ extracted the variables statistically useful for prediction. The Boruta algorithm evaluates the importance of variables by comparing them to importance achieved at random, thereby reducing the number of analyzed variables to create a simpler model with good performance. Using variables extracted in 101 models or more, we again performed a repeated (100 × 4 times) nested cross‐validation strategy classification with XGBoost (namely “final modeling”).

The final model's performance was evaluated by the mean of the area under the receiver operating characteristic curve (ROC AUC) across all the 400 models. AUC ratings are: Excellent (≥0.9), Good (≥0.8), Fair (≥0.7), Poor (≥0.6), and Fail (≥0.5).^37^ For model explainability, Shapley additive explanations (SHAP)^38^ were used to compute the magnitude and direction of influence of each variable on model output. SHAP values take into account the interaction between variables, meaning that the contribution of any single variable is evaluated in the context of changes in other variables. SHAP values were represented as averages across all 400 models.

Data analysis and modeling were performed using Python 3.9.13. Main libraries were Pandas and Numpy for data manipulation, BorutaPy for feature selection, Scikit‐learn and XGBoost for modeling, SHAP for calculating feature importance, and Matplotlib and Seaborn for plotting.

## RESULTS

### Demographic and clinical characteristics

**Figure 1:**
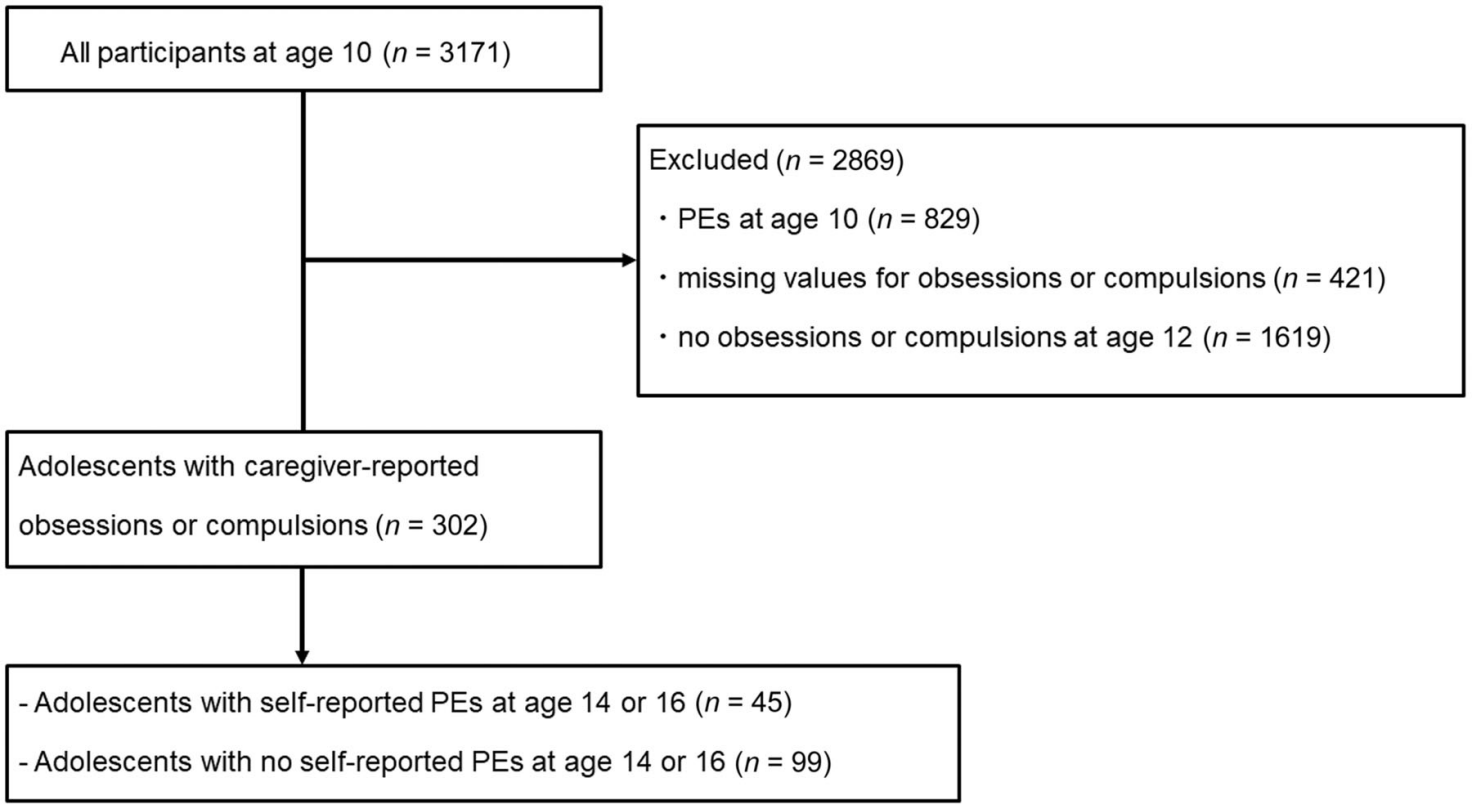
Participant flow chart. Data on the 144 participants with no psychotic experiences (PEs) at Time Point 1 and obsessive and compulsive symptoms at Time Point 2 were analyzed.

One hundred and forty‐four participants did not have PEs at T1 and had OC symptoms at T2. Data on these 144 participants were analyzed (Figure 1). While 45 participants had PEs at T3 or T4, 99 had no PEs at T3 or T4. OC symptoms at T2 were more common in boys (83) than girls (61). The characteristics of the adolescents with or without PEs at T3 or T4 are presented in Table 1.

**Table 1:** Demographic and clinical characteristics of study participants.

			All participants at age 10		Participants without PEs at age 10 and with OC symptoms at age 12			
					PEs at age 14 or 16			
					PE (−)		PE (+)
					*n* = 99		*n* = 45	
			Mean	SD	Mean	SD	Mean	SD
BMI	Age 10		16.8	(2.3)	16.3	(1.7)	17.0	(2.2)
IQ	Age 10		107.7	(14.1)	110.5	(13.9)	108	(15.6)
AQ	Age 12		2.3	(1.9)	3.2	(1.8)	3.1	(2.0)
Child's time discount	Age 10		**−**5.3	(2.4)	**−**5.4	(2.2)	**−**5.5	(2.2)
Caregiver's time discount	Age 10		**−**5.6	(2.4)	**−**5.4	(1.9)	**−**6.0	(1.6)

			*n*	(%)	*n*	(%)	*n*	(%)
Sex		Girls	1684	(53.1%)	38	(38.4%)	23	(51.1%)
		Boys	1487	(46.9%)	61	(61.6%)	22	(48.9%)
Bullied	Age 10	Bullied	175	(5.5%)	24	(24.2%)	25	(55.6%)
		Not bullied	2953	(93.1%)	75	(75.8%)	20	(44.4%)
Family income	Age 10	0–4.9	617	(19.4%)	16	(16.2%)	9	(20.0%)
[million yen/year]		5.0–9.9	1507	(47.5%)	58	(58.6%)	23	(51.1%)
		10.0+	916	(28.9%)	25	(25.3%)	13	(28.9%)
Obsession	Age 10	No	2480	(78.2%)	53	(53.5%)	21	(46.7%)
		Somewhat true	584	(18.4%)	42	(42.4%)	21	(46.7%)
		True	94	(3.0%)	4	(4.0%)	3	(6.7%)
Compulsion	Age 10	No	3093	(97.5%)	94	(94.9%)	42	(93.3%)
		Somewhat true	54	(1.7%)	5	(5.1%)	2	(4.4%)
		True	13	(0.4%)	0	(0.0%)	1	(2.2%)
Obsession	Age 12	No	2288	(72.2%)	4	(4.0%)	0	(0.0%)
		Somewhat true	410	(12.9%)	89	(89.9%)	39	(86.7%)
		True	52	(1.6%)	6	(6.1%)	6	(13.3%)
Compulsion	Age 12	No	2710	(85.5%)	91	(91.9%)	44	(97.8%)
		Somewhat true	40	(1.3%)	8	(8.1%)	1	(2.2%)
		True	5	(0.2%)	0	(0.0%)	0	(0.0%)
Obsession	Age 16	No	1758	(55.4%)	55	(55.6%)	20	(44.4%)
		Somewhat true	428	(13.5%)	41	(41.4%)	18	(40.0%)
		True	47	(1.5%)	3	(3.0%)	4	(8.9%)
Compulsion	Age 16	No	2177	(68.7%)	94	(94.9%)	38	(84.4%)
		Somewhat true	51	(1.6%)	5	(5.1%)	4	(8.9%)
		True	7	(0.2%)	0	(0.0%)	0	(0.0%)

Abbreviations: AQ, autism‐spectrum quotient; BMI, body mass index; IQ, intelligence quotient; OC, obsessive–compulsive; PEs, psychotic experiences; SD, standard deviation.

### Predictors for PEs at T3 or T4

**Figure 2:**
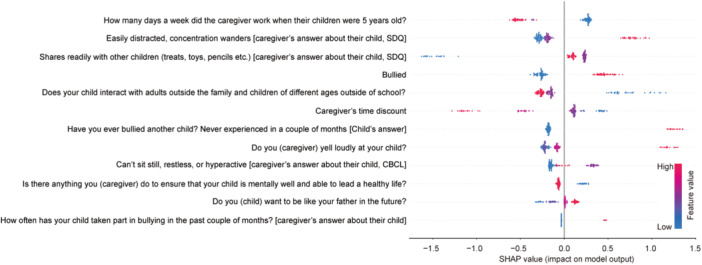
Shapley additive explanations (SHAP) analysis of variables related to psychotic experiences (PEs) at Time Point (T)3 or T4. SHAP values for each participant were plotted against 12 variables extracted by the Boruta algorithm. The color indicates the value or the category of the answer (blue if low, red if high in quantitative question; blue if negative, red if positive answer in categorical question). The higher SHAP value indicates a higher degree of prediction of PEs at T3 or T4. CBCL, Child Behavior Checklist; SDQ, Strengths and Difficulties Questionnaire.

The Boruta algorithm reduced from an initial 630 variables to 12 important and relevant variables. The average SHAP values (importance and directionality on the predicting model) of each variable are shown in Figure 2.

The baseline participant characteristics predicting PEs at T3 or T4 were “bullying victimization at school in the past couple of months,” “not having opportunities to interact with adults outside the family and children of different ages in activities outside of school,” “being easily distracted, concentration wanders,” “sometimes being hyperactive, or not being capable of sitting still,” “bullying perpetration (reported either by participants themselves or by their caregivers),” and “having the desire to be like their father in the future.” On the other hand, “not sharing readily with other children (treats, toys, pencils, etc.)” predicted no PEs at T3 and T4.

Primary caregivers' characteristics predicting PEs included “working 0 days in a week when their children were 5 years old,” “having smaller time discount (offering subjects a series of choices between immediate rewards and larger, delayed rewards),”^39^ “always yelling loudly at their children,” and “not doing anything to ensure that their child would lead a healthy life.”

**Figure 3:**
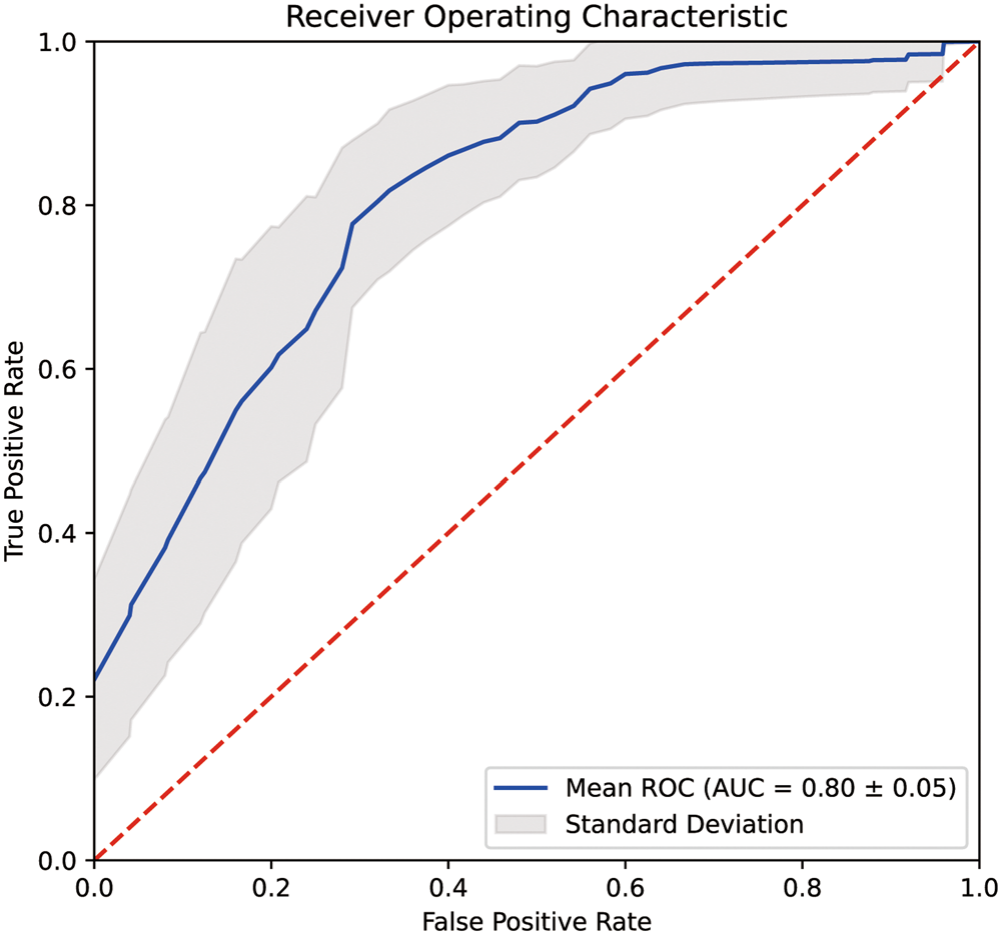
Out‐of‐fold model performance. Blue line represents an averaged receiver operating characteristic (ROC) curve depicting predictive performance on unseen out‐of‐fold data across all 400 models. The gray area represents 1 standard deviation. AUC, area under the curve.

The final modeling using the 12 variables demonstrated a “Good” ability to distinguish those who had PEs from those who had no PEs at T3 and T4. Out‐of‐fold AUC ± standard deviation (SD) was 0.80 ± 0.05 (Figure 3).

## DISCUSSION

Using data from a population‐based longitudinal cohort study, we developed an algorithm demonstrating a “Good” ability to predict the emergence of PEs in adolescents with OC symptoms. Twelve variables, including psychosocial factors, were identified as useful for predicting subsequent PEs in adolescents with OC symptoms.

### Novel predictors for PEs among adolescents with OC symptoms

As far as we know, previous literature has not reported the following variables as predictors for PEs. They can be categorized as caregiver–child relationships and interpersonal relationships with others outside of the family.

#### Caregiver–child relationship

According to our model, increased proximity to family members might be implicated in increased risk of PEs. Predictors of subsequent PEs in adolescents with OC symptoms included having a desire to be like their father in the future and nonworking of primary caregiver when they were 5 years old. These findings may look inconsistent with the prior study suggesting that higher positive parent‐child relationship scores were associated with reduced risk of PEs in the general population.^40^ However, a child's desire to be like their father might not always reflect a positive parent–child relationship, but rather a pressure to be like their father. A previous study indicated that individuals with subclinical OC symptoms perceived their parents as overprotective, and fathers as more perfectionistic and critical.^41^

#### Relationships with others outside of family members

In terms of interpersonal relationships outside the family, PEs were predicted by limited interaction with people of different ages. The relationships with adults outside the family would be protective, which is in line with the previous studies. Promoting positive relationships with nonfamilial adults in childhood may lead to reduced distress in the long term.^42^ Limited relationships with adults outside the family might increase a feeling of loneliness, which was a risk for PEs.^43^, ^44^

### Predictors for no PEs

Not readily sharing their belongings (e.g., pencils) with other children was a strong predictor of having no PEs in later adolescence. This behavior may be associated with contamination fear, which is characteristic of contamination‐related OCD and is distinct from psychotic delusions.^45^

### Predictors consistent with risk factors for PEs in the general populations

Several predictors for PEs extracted in this study were consistent with known risk factors for PEs in the general population,^46^ including trauma, externalizing problems, and attention deficit hyperactivity symptoms.

Bullying victimization at school and strict parenting styles (i.e., “always yelling loudly at their children” and “not doing anything to ensure that their child would lead a healthy life”) were the predictors for PEs. Previous research indicated that childhood trauma (bullying and physical assault) predicted PEs at 12 months ahead.^47^ Our study suggests that adolescents with OC symptoms also have higher risk of developing PEs when they have undergone harsh parenting or deficient warmth in caregiving in their earlier life.

Bullying perpetration was reported more among the participants with subsequent PEs in this study. Previous studies indicated that such externalizing problems were associated with an increased risk of PEs.^48^

The group with PEs at T3 or T4 was also characterized by attention deficit hyperactivity symptoms (i.e., “being easily distracted, concentration wanders” and “sometimes being hyperactive, or not being capable of sitting still”). This finding is in line with a previous study showing the relation between attention‐deficit/hyperactivity disorder (ADHD) and later PEs.^49^ Conversely, the identified predictors included caregivers’ smaller time discounting, which is inconsistent with the literature suggesting that PEs were associated with higher time discounting.^50^

### Clinical implications and for future studies

This study could contribute to predicting subsequent PEs among adolescents with OC symptoms. We provided data‐driven speculation that close‐knit family bonds and limited social connections outside the family may predict later PEs. When supporting adolescents with OC symptoms, one should assess the relationships both outside and inside the family circle, as well as the known risk factors for PEs (for instance, trauma, externalizing problems, and ADHD symptoms). Even if an individual is predicted to experience subsequent PEs, pharmacological approaches (e.g., antipsychotics and SSRIs) are controversial due to concerns over side‐effects. Instead, easily accessible youth mental health centers have been increasingly recommended, offering services for a wide range of mental health conditions.^51^ Further validation in a clinical setting is required to elucidate the mechanisms underlying these findings for early intervention strategies. For example, the variables extracted in this study could be used in follow‐up studies of people who had OC symptoms at the first visit. This may provide insights into the selection of intervention methods, such as pharmacological treatment or referral to a youth mental health center. In addition, we are going to collect data from this cohort into their twenties and beyond, identifying the trajectories of the extracted predictors themselves at later time points.

### Strengths and limitations

Our study has several strengths. We used data from a longitudinal cohort from the general population, enabling us to approach the group with subclinical symptoms. We could explore predictor candidates widely by using 630 variables at T1. Bias was minimized by extensive validation procedures across the 400 unique models.

This study has some limitations. First, there is a risk of overfitting due to the small number of participants. For reducing the risk of overfitting, a grid search was applied for tuning hyperparameters via repeated nested cross‐validation. Second, generalizability of our results to the population outside Japan remains to be elucidated. Third, SHAP values are typically used to assess relative importance within the same model, but SHAP values cannot reflect the absolute effect size of each predictor. Fourth, since PEs at T2 were not considered for an exclusion criterion, it cannot be strictly said that OCs preceded PEs in the included participants. Fifth, we acknowledge the inability to make causal interpretations is one of the most substantial limitations of this paper from a methodological perspective. It is important to note that machine learning models are designed for prediction and are not suitable for causal interpretation.^52^

## CONCLUSION

Close‐knit family bonds and limited social connections outside the family predict later PEs among adolescents with OC symptoms.

## AUTHOR CONTRIBUTION

Yutaka Sawai has full access to all data and takes responsibility for its integrity and the accuracy of the data analysis. Yutaka Sawai and Shuntaro Ando contributed to the study concept and design. Yutaka Sawai, Riki Tanaka, Rin Minami, Daiki Nagaoka, Akito Uno, Ayako Okuma, Syudo Yamasaki, Mitsuhiro Miyashita, Atsushi Nishida, Kiyoto Kasai, and Shuntaro Ando contributed to data acquisition and interpretation. Yutaka Sawai performed the data and statistical analyses. Yutaka Sawai drafted the manuscript. Yutaka Sawai, Riki Tanaka, Rin Minami, Daiki Nagaoka, Akito Uno, Ayako Okuma, Syudo Yamasaki, Mitsuhiro Miyashita, Atsushi Nishida, Kiyoto Kasai, and Shuntaro Ando contributed to the critical revision of the manuscript for important intellectual content.

## CONFLICT OF INTEREST STATEMENT

The authors declare no conflict of interest.

## ETHICS APPROVAL STATEMENT

The protocol for the research project has been approved by a suitably constituted Ethics Committee of three research institutes: Tokyo Metropolitan Institute of Medical Science (approval No. 12–35), the University of Tokyo (10057), and the Graduate University for Advanced Studies (2012002). It conforms to the provisions of the Declaration of Helsinki. Written informed consent was obtained from the children's primary caregivers during each wave of the study, and their anonymity was preserved.

## PATIENT CONSENT STATEMENT

N/A

## CLINICAL TRIAL REGISTRATION

N/A

## Data Availability

Collaboration in data analysis and publication will be welcome through specific research proposals sent to the research committee. The initial contact point for collaborations is [nishida-at@igakuken.or.jp].
